# Bisphenol A Exacerbates Allergic Inflammation in an Ovalbumin-Induced Mouse Model of Allergic Rhinitis

**DOI:** 10.1155/2020/7573103

**Published:** 2020-09-08

**Authors:** Yunxiu Wang, Zhiwei Cao, He Zhao, Yaoyao Ren, Liying Hao, Zhaowei Gu

**Affiliations:** ^1^Department of Otolaryngology Head and Neck Surgery, Shengjing Hospital of China Medical University, Shenyang City, 110004 Liaoning Province, China; ^2^Department of Pharmaceutical Toxicology, School of Pharmacy, China Medical University, Shenyang City, 110122 Liaoning Province, China

## Abstract

**Purpose:**

Bisphenol A (BPA) is found in many plastic products and is thus a common environmental endocrine disruptor. Plastic-related health problems, including allergic diseases, are attracting increasing attention. However, few experimental studies have explored the effect of BPA on allergic rhinitis (AR). We explore whether BPA was directly related to the allergic inflammation induced by ovalbumin (OVA) in AR mice.

**Methods:**

We first constructed OVA-induced mouse model, and after BPA administration, we evaluated nasal symptoms and measured the serum OVA-specific IgE levels by ELISA. Th2 and Treg-related cytokines of nasal mucosa were measured by cytometric bead array. Th2 and Treg-specific transcription factor levels were assayed by PCR. The proportions of CD3^+^CD4^+^IL-4^+^Th2 and CD4^+^Helios^+^Foxp3^+^ T cells (Tregs) in spleen tissue were determined by flow cytometry.

**Results:**

Compared to OVA-only-induced mice, BPA addition increased nasal symptoms and serum OVA-specific IgE levels. OVA and BPA coexposure significantly increased IL-4 and IL-13 protein levels compared to those after OVA exposure alone. BPA plus OVA tended to decrease the IL-10 protein levels compared to those after OVA alone. Coexposure to OVA and BPA significantly increased the GATA-3-encoding mRNA level, and decreased the levels of mRNAs encoding Foxp3 and Helios, compared to those after OVA exposure alone. BPA increased the Th2 cell proportion, and decreased that of Tregs, compared to the levels with OVA alone.

**Conclusion:**

BPA exerted negative effects by exacerbating AR allergic symptoms, increasing serum OVA-specific IgE levels, and compromising Th2 and Treg responses.

## 1. Introduction

Allergic rhinitis (AR) is feature with nasal itching, sneezing, watery secretions, and congestion, reflecting the IgE-mediated mucosal inflammation driven by Th2 cells. AR affects over 500 million people worldwide [1, 2]. Regulatory T cells (Tregs) play an important role in preventing Th2-mediated inappropriate responses to environmental allergens [3]. In recent years, the morbidity rate of AR has raised, especially among preschool children [4–6]. However, whether there is any role for external factors remains unclear. Bisphenol A (BPA) is a common environmental endocrine disruptor, being widely found in plastics. Humans come into contact with BPA via the skin and when consuming food and water packaged in plastic containing BPA [7]. BPA is an endocrine disruptor and may act as a weak estrogen; public health problems associated with BPA have attracted increasing attention [8]. BPA exposure during the perinatal or prenatal period exacerbated allergic sensitization and bronchial inflammation in asthma model [9, 10], and phthalates and BPA exacerbated atopic dermatitis in children [11]. Although epidemiological studies have not yet clearly shown that BPA increases the incidence rates of allergy and asthma, it may enhance the risk of a Th2 response by altering immune cell function and cytokine production [12]. BPA combined with OVA exacerbated eosinophilia severity in the lungs of adult mice, perhaps by promoting a Th2-biased immune response [13]. As far as we know, few experimental studies have explored the effect of BPA on AR. Here, we explore whether BPA is directly related to the allergic inflammation induced by ovalbumin (OVA) in AR mice.

## 2. Materials and Methods

### 2.1. Reagents

RPMI 1640 was from Gibco, Carlsbad, CA. A Total RNA Extraction Kit was obtained from Solarbio (Beijing, China). BPA and OVA were obtained from Sigma-Aldrich (St. Louis, MO). A PrimeScript RT kit was ordered from Takara (Dalian, China). The APC-Cy7-CD3, FITC-CD4, PE-IL-4, PE-Foxp3, APC-Helios, and CBA Flex Set were obtained from BD Biosciences (Franklin Lakes, NJ).

### 2.2. AR Murine Model and BPA Intervention

**Figure 1:**
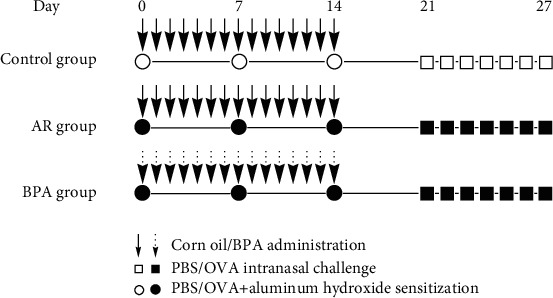
The experimental protocol. OVA sensitization was followed by OVA challenge to trigger AR development. Briefly, on days 0, 7, and 14, mice in the OVA and BPA groups were sensitized with 2 mg of aluminum hydroxide (100 *μ*L of solution) and OVA (100 *μ*g) via intraperitoneal injection; control mice received PBS alone. Mice in the BPA group were subcutaneously injected with 0.5 mg/kg/day BPA in corn oil on days 0–14; the other two groups received only corn oil. The OVA and BPA groups were intranasally challenged with 100 *μ*g OVA in 20 *μ*L PBS on days 21–27. The control group received PBS alone. OVA: ovalbumin; AR: allergic rhinitis; BPA: bisphenol A; PBS: phosphate-buffered saline.

BALB/c mice (8 weeks, female) were purchased from Changsheng Biotechnology Co., Ltd., Liaoning, China. All mice were raised on an OVA-free diet and randomly assigned to control, AR, and BPA groups (*n* = 10 each). The AR model has been described previously [14]. On day 0 to day 14, BPA mice were subcutaneously injected with 0.5 mg/kg/d of BPA in corn oil; the other two groups received only corn oil. The experimental protocol, shown in Figure 1, was approved by the Ethics Committee of Shengjing Hospital.

### 2.3. Evaluation of Nasal Symptoms and Sample Collection

After the last intranasal OVA challenge, the numbers of sneezes and nose rubs over 15 min were recorded. Blood samples were collected from mice that were sacrificed under anesthesia; serum was obtained via centrifugation and stored at −80°C prior to IgE detection. Nasal mucosal samples were stored for cytokine and quantitative real-time PCR (qRT-PCR) assays. Spleens were removed for detection of CD3^+^CD4^+^IL-4^+^ Th2 and CD4^+^Helios^+^ Foxp3^+^ Tregs via flow cytometry.

### 2.4. Cytokine Measurements and Detection of OVA-Specific IgE

Nasal mucosa samples were crushed and centrifuged, and supernatant IL-4, IL-5, IL-13, and IL-10 levels were assayed using the CBA Flex Set. All samples underwent flow cytometry using the FACS Aria III instrument (BD Biosciences); the data were processed using FACSDiva and BD CBA software ver. 4.2 (BD Biosciences). Serum OVA-specific IgE levels were determined by ELISA kits (BioLegend).

### 2.5. qRT-PCR Analysis of Nasal Mucosal Samples

Total RNA was collected using the Total RNA Extraction Kit, and complementary DNA (cDNA) was synthesized via reverse transcription using the PrimeScript RT kit according to the manufacturer's instructions. qRT-PCR was performed using a Roche LightCycler 480 II system (Roche, Basel, Switzerland). The sequences of the PCR primers were listed in Table 1.

The relative expression of the three target genes was determined using the cycle threshold (2^-*△△*CT^) method and normalized to the *β*-actin level.

### 2.6. Detection of CD3^+^CD4^+^IL-4^+^Th2 and CD4^+^Helios^+^Foxp3^+^Tregs via Flow Cytometry

Splenic mononuclear cells were placed in tubes containing RPMI 1640 medium; CD3^+^CD4^+^IL-4^+^Th2 and CD4^+^Helios^+^Foxp3^+^Tregs were detected as described previously [15]. Stained cells were washed once and subjected to FACS Aria III flow cytometry (BD Biosciences); the results were analyzed using FlowJo software (ver. 7.6; TreeStar Inc., Ashland, OR).

### 2.7. Statistical Analysis

All data are expressed as means ± SEM. One-way ANOVA was used to compare the groups. A *P* value < 0.05 was taken to indicate statistical significance. GraphPad Prism software (GraphPad Software Inc., La Jolla, CA) was used to statistically analyze and draw graphs.

## 3. Results

### 3.1. Effects of BPA on OVA-Induced AR Nasal Symptom and OVA-Specific IgE Levels

**Figure 2:**
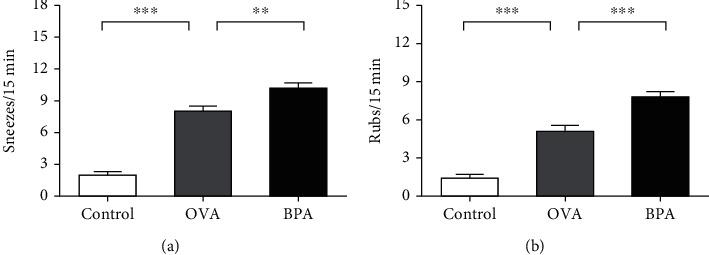
The numbers of (a) sneezes and (b) nasal rubs for each mouse were counted over 15 min from the last intranasal OVA challenge. Data are expressed as means ± SEM; *n* = 10 in each group. ^∗∗^*P* < 0.01; ^∗∗∗^*P* < 0.001.

**Figure 3:**
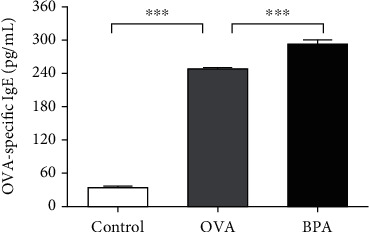
BPA increased the serum OVA-specific IgE levels, as shown by ELISA. Data are expressed as means ± SEM; *n* = 5 in each group. ^∗∗∗^*P* < 0.001.

Sneezing and nose scratching are the principal symptoms of AR; any effect of BPA on AR depends on the extent to which BPA affects these symptoms. We recorded numbers of sneezes and nose rubs in the three groups of mice over 15 min after the last intranasal OVA challenge. As shown in Figure 2, the OVA and BPA groups exhibited significantly more symptoms than the control (*P* < 0.05); OVA plus BPA mice (BPA group) showed more symptoms than the OVA-only group (OVA group) (*P* < 0.05). Serum OVA-specific IgE levels were significantly elevated in the OVA-induced group compared to the control group (*P* < 0.05) and were even higher in the OVA plus BPA group (Figure 3). BPA aggravated AR nasal symptoms and serum OVA-specific IgE levels.

### 3.2. Effects of BPA on Th2 Cytokine Levels in Nasal Mucosa

**Figure 4:**
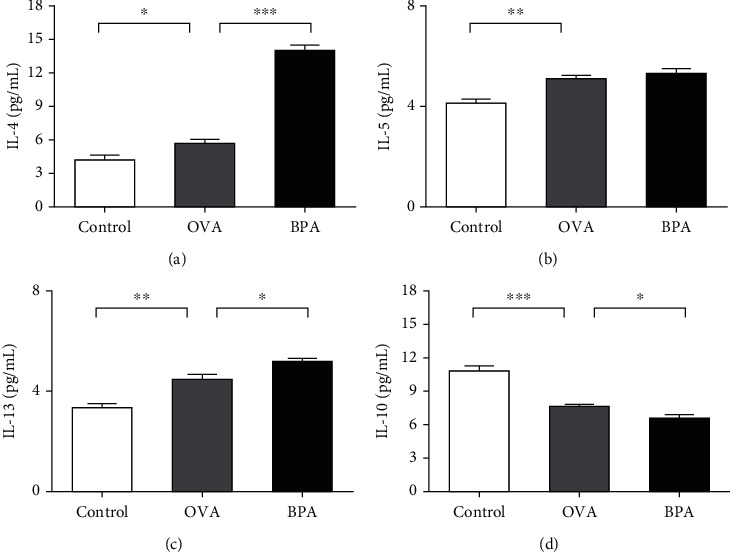
BPA increased the levels of Th2 cell effector cytokines and repressed cytokine expression by Tregs in the nasal mucosa. The protein expression levels of (a) IL-4, (b) IL-5, (c) IL-13, and (d) IL-10 were measured using the CBA Flex Set (BD Biosciences). Data are expressed as means ± SEM; *n* = 5 in each group. ^∗^*P* < 0.05; ^∗∗^*P* < 0.01; ^∗∗∗^*P* < 0.001.

Th2-mediated cytokines cause inflammation in AR. The effects of BPA on nasal mucosal Th2 cytokine levels after the last intranasal OVA challenge were investigated. Compared to PBS, OVA increased IL-4, IL-5, and IL-13 protein levels (*P* < 0.05, Figures 4(a)–4(c)). Compared to OVA alone, BPA further increased IL-4 and IL-13 protein levels (*P* < 0.05, Figures 4(a) and 4(c)). BPA also increased the IL-5 levels, but not significantly (Figure 4(b)).

### 3.3. Effect of BPA on the Cytokine Levels of Tregs

As we knew, IL-10, a major cytokine of Tregs, plays an important role in the development of AR.  Figure 4(d) shows that, compared to PBS, OVA reduced IL-10 protein levels (*P* < 0.05). BPA tended to further reduce the protein levels (*P* < 0.05).

### 3.4. Effect of BPA on Th2 Cell-Specific Transcription Factors

**Figure 5:**
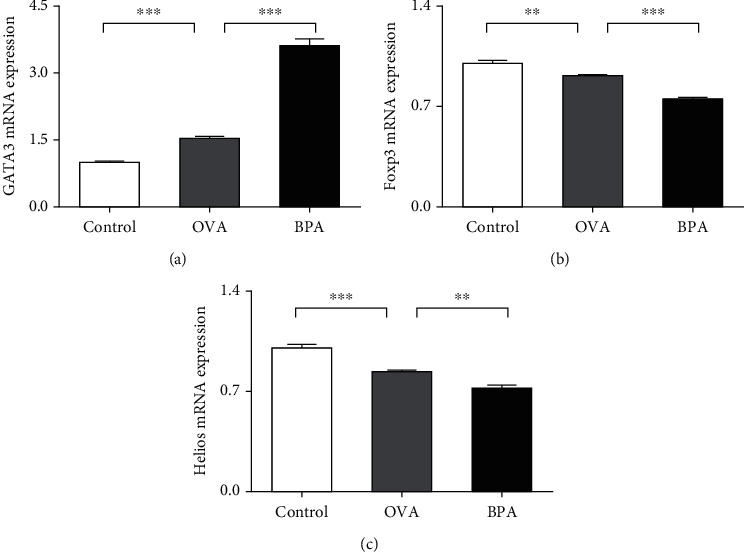
BPA increased the levels of the Th2 cell-specific transcription factor GATA-3 and reduced those of the Treg-specific factors Foxp3 and Helios. RT-PCR data on (a) GATA-3, (b) Foxp3, and (c) Helios mRNA expression levels in nasal mucosa. Data are expressed as means ± SEM; *n* = 5 in each group. ^∗∗^*P* < 0.01; ^∗∗∗^*P* < 0.001.

The effect of BPA on the levels of a Th2 cell-specific transcription factor (GATA-3) was evaluated. Compared to PBS, OVA increased the levels of mRNA encoding GATA-3 (*P* < 0.05, Figure 5(a)). BPA further increased the levels (*P* < 0.05).

### 3.5. Effect of BPA on Treg-Specific Transcription Factor Levels

Foxp3 is the most specific marker of Tregs; Helios status is helpful for identifying Treg subsets showing consistent suppressive activity [16]. We measured the levels of mRNA encoding Foxp3 and Helios. Figures 5(b) and 5(c) show that, compared to PBS, OVA reduced these levels; BPA further reduced the levels (*P* < 0.05).

### 3.6. Effect of BPA on the Proportions of Th2 Cells

**Figure 6:**
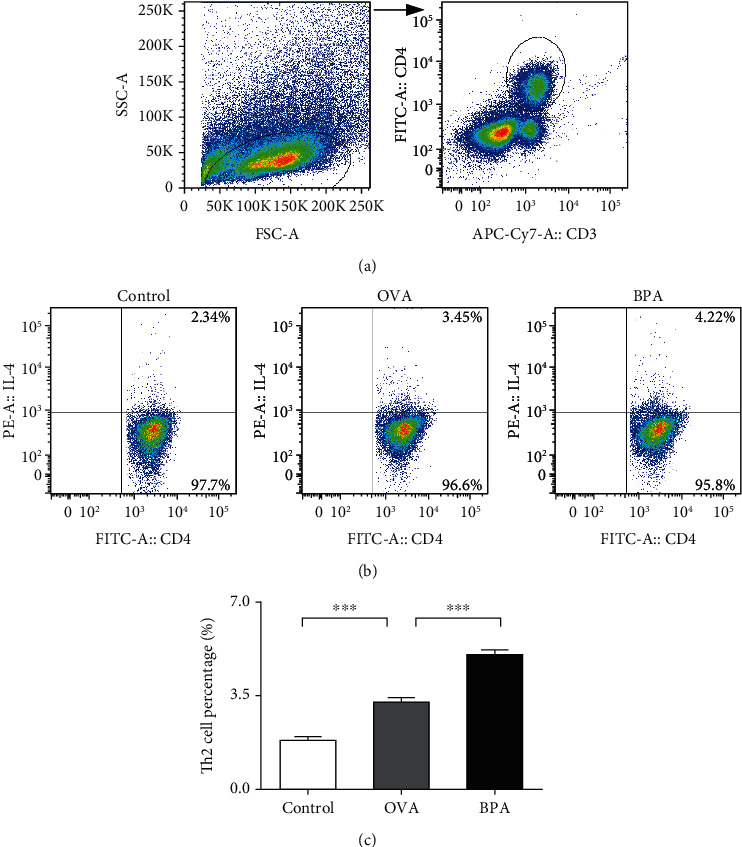
Flow cytometry showed that BPA increased the proportions of Th2 cells in the spleens of mice with OVA-induced AR. CD3^+^CD4^+^ T cell subgroup (a). Representative staining of CD3^+^CD4^+^ IL-4^+^Th2 cells of each group. The numbers in the upper right quadrants are the proportions of Th2 cells (b). The statistical data (c). Data are expressed as means ± SEM; *n* = 5 in each group. ^∗∗∗^*P* < 0.001.

An imbalance among the CD4^+^ Th cell subsets, particularly Th2 cells, triggers and maintains allergic responses [17]. We assessed the effect of BPA on the proportions of Th2 cell (Figure 6). OVA increased the proportions of CD3^+^CD4^+^IL-4^+^Th2 cells compared to control mice; BPA further increased the proportions of these cells (*P* < 0.05).

### 3.7. Effect of BPA on the Proportion of Tregs

**Figure 7:**
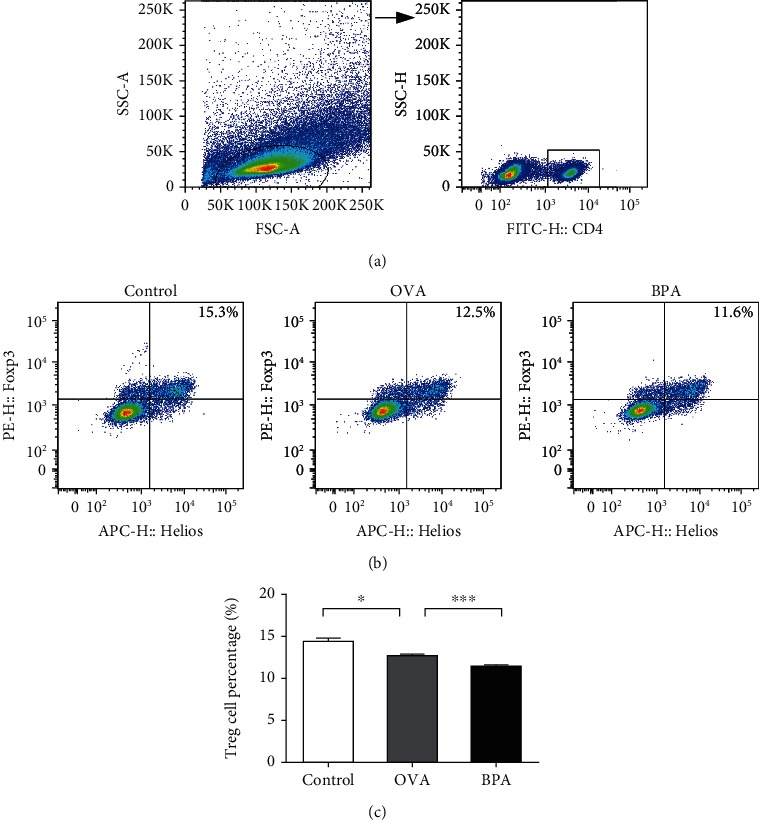
Flow cytometry showed that BPA reduced the proportion of Tregs in spleens of mice with OVA-induced AR. CD4^+^ T cells (a). Representative staining of CD4^+^Helios^+^Foxp3^+^Tregs from each group. The numbers in the upper right quadrants are the proportions of Tregs (b). The statistical data (c). Data are expressed as means ± SEM; *n* = 5 per group. ^∗^*P* < 0.05; ^∗∗∗^*P* < 0.001.

Tregs modulate the immune system and maintain tolerance to self-antigens [18]. We measured the proportion of CD4^+^Helios^+^Foxp3^+^Tregs via flow cytometry; compared to PBS, OVA decreased the proportions of these cells (*P* < 0.05, Figure 7). BPA further decreased the proportions of the cells compared to OVA alone (*P* < 0.05).

## 4. Discussion

We used an established OVA-induced AR murine model to explore whether BPA affected allergic reactions. Nasal symptoms were exacerbated; meanwhile, OVA-specific IgE levels were increased, in BPA plus OVA-treated AR mice. BPA increased the proportions of Th2 cells, as well as the mRNA levels of GATA-3- and Th2-related cytokines IL-4 and IL-13. BPA downregulated Treg cells, the mRNA levels of Helios and Foxp3, and Treg-related cytokine IL-10. Many previous studies have focused on off-target effects of BPA [19]; we evaluated the direct effects of BPA on allergic inflammation. Mice with AR received BPA prior to OVA challenge; we sought to establish if there was a direct relationship between the allergic response and BPA. Allergens activate the binding of IgE to Fc*ε*RI located on the surface of eosinophils; chemical mediators are then released, triggering clinical symptoms of allergy [17]. AR symptoms are thus attributable to IgE-mediated inflammation of the nasal mucosa. We found that BPA exposure significantly increased allergic symptoms and serum OVA-specific IgE levels; thus, BPA directly affected experimental AR.

AR is a common disorder caused by an inappropriate Th2-mediated immune response to environmental antigens [20]. Th2 cells secrete specific cytokines, including IL-4, IL-5, and IL-13 [21–23], which are important drivers of AR immunopathology [24]. IL-4 promotes T cell activation and differentiation into Th2 cells; IL-4 and IL-13 play roles in B cell differentiation and IgE and mucus production in the airway [25–27]. IL-5 is locally produced at sites of allergic inflammation and recruits eosinophils from the bone marrow; these cells contribute to AR injury by releasing cytotoxic granular proteins [22, 28]. The GATA-3 transcription factor is specific to Th2 cells. It promotes the production of IL-4, IL-5, and IL-13; induces Th0 to differentiate into Th2; and inhibits Th1 cell differentiation [29]. We explored the effects of BPA on Th2 cells by measuring the proportions of CD3^+^CD4^+^IL-4^+^Th2 cells, as well as the protein levels of Th2-related cytokines, and the mRNA level of GATA-3. BPA significantly increased expression of all of these cells and factors. Our results are similar to those of Yanagisawa [30]; OVA and BPA coexposure increased the mouse lung levels of mRNAs encoding IL-4, IL-5, and IL-13 compared to those after OVA exposure alone [30]. We found that the IL-5 level in the BPA mice was somewhat higher than in the OVA ones; however, the difference was not significant, perhaps because of the small sample sizes (*n* = 5/group).

Treg prevents inappropriate Th2 responses to environmental allergens [3, 31]. Th2 cells secrete inhibitory cytokines, including IL-10, that induce Treg formation from naive T cells and inhibit the development of other types of immune cells [32]. Foxp3 is a Treg-related transcription factor, and Helios is a marker of Treg activation [33, 34]. The effects of BPA on the Treg response were evaluated by measuring the proportion of Tregs, IL-10 protein levels, and Helios and Foxp3 mRNA levels. Compared to OVA alone, coexposure to OVA and BPA reduced the Treg response (i.e., the proportion of Tregs, IL-10 protein levels, Helios and Foxp3 mRNA levels). Previous studies showed that BPA reduced the Tregs proportion, thus compromising the immune system; the Th1/Th2 ratio changed and disease developed [35, 36].

BPA is a common environmental endocrine disruptor, which can disrupt the human endocrine, reproductive, and immune systems through cell signaling pathways, and can increase the risk for certain diseases, including obesity, cardiovascular disease, brain disease, asthma, and even cancer [36, 37]. The signaling pathways affected by BPA include signal transducer and activator of transcription 3 (STAT3), early growth response gene-2, NF-*κ*B, and ERK1/2 [38–43].

Previous AR immune cellular signal pathway studies have mainly focused on the influence of the STAT family on Th differentiation [44]. STAT family members include STAT1, STAT2, STAT3, STAT4, STAT5A, STAT5b, and STAT6 [45]. STAT1 and STAT4 are the key factors of IFN-*γ* signaling and IL-12 signaling, respectively, and both are critical for Th1 polarization [46]. However, STAT6 can inhibit Th1 polarization, which is an important factor in Th2 signal transduction [46]. STAT5 can regulate the differentiation of Treg cells by regulating Foxp3 expression [47]. STAT3 is an essential transcription factor for Th17 differentiation and Treg inhibition [45, 48]. In addition, it can promote the development of Th2 cells under the background of STAT6 signaling [49].

STAT3 is activated by immune cytokines and endocrine-disrupting chemicals, including BPA [50–52]. Therefore, we speculated that STAT3 may be activated after exposure to BPA, the Th2 response is enhanced, and Treg reaction is inhibited through the STAT3 signaling pathway, further aggravating AR inflammation.

Our previous studies confirmed the effects of the environmental hormone nonylphenol on AR, and showed that nonylphenol can aggravate Th2-associated immune reactions in an AR mouse model [14]. The present study focused on BPA. However, a comprehensive analysis is required to determine the combined actions of environmental endocrine disruptors.

## 5. Conclusion

Our findings provide evidence of the negative effects of BPA in an OVA-induced AR mouse model. BPA can exacerbate AR allergic symptoms, increase serum levels of OVA-specific IgE, and compromise Th2 and Treg responses. Our results serve as a warning regarding the adverse effects of BPA in adult AR.

## Figures and Tables

**Table 1:** Primer sequences for quantitative real-time PCR.

Gene	Sense	Antisense
*β*-Actin	GCAGAAGGAGATTACTGCTCT	GCTGATCCACATCTGCTGGAA
Gata3	TACCACCTATCCGCCCTATG	GCCTCGACTTACATCCGAAC
Foxp3	GCCAAGCAGAAAGATGACAG	TTCCAGATGTTGTGGGTGAG
Helios	GGTACCGATGTGCTCTGCCT	TGTCCCTGTCACAGCAGAGC

## Data Availability

The data used to support the findings of this study are available from the corresponding author upon request.
